# New PET/CT Features for the Evaluation of Tumor Response

**DOI:** 10.4172/2155-9619.1000e110

**Published:** 2013-07-25

**Authors:** Wei Lu

**Affiliations:** Department of Radiation Oncology, University of Maryland School of Medicine, Baltimore, USA

## Abstract

With the emerging multi-modality imaging performed at multiple time points for each patient, it becomes more important to analyze the serial images quantitatively, select and combine both complementary and contradictory information from various sources, for accurate and personalized evaluation of tumor response to therapy.

## CT Features

Assessment of the change in tumor burden or tumor response is an important feature of the clinical evaluation of cancer therapeutics [1]. Traditionally, response of solid tumors to cancer therapy is evaluated visually or measured with anatomic changes in tumor diameters using CT imaging according to the Response Evaluation Criteria in Solid Tumors (RECIST) or World Health Organization (WHO) criteria [1–3]. Briefly, RECIST [1] defines complete response (CR) as disappearance of all target lesions, partial response (PR) as at least 30% decrease in the sum of (largest) diameters (in the axial plane) of target lesions from the baseline, progressive disease (PD) as at least 20% increase and an absolute increase of at least 5 mm in the sum of diameters, and stable disease (SD) as neither sufficient shrinkage to qualify for PR nor sufficient increase to qualify for PD.

Recent studies show that new CT features, including volumetric, attenuation, morphologic, structure, and texture descriptors, have advantages over the RECIST and WHO criteria in certain tumor types. Both RECIST and WHO criteria are linear measurements of tumor size, which have limitations related to technical variability, tumor morphology, and reader decisions. With the thin-section CT, it is possible to measure tumor volume using segmentation methods with adequate spatial resolutions [4,5], which overcomes some of the limitations of linear measurements. Changes in attenuation in contrast-enhanced CT (CECT) has been shown correlate better with response than changes in tumor size in hepatocellular carcinoma [6] and gastrointestinal stromal tumor [7]. One advantage of attenuation features is that they can take into consideration of tumor necrosis [6]. In colorectal liver metastases, morphologic evaluation based on metastases changing from heterogeneous masses into homogeneous hypoattenuating lesions had a statistically significant association with pathologic response and survival while RECIST did not [8]. Adding structure features (presence or absence of marked central necrosis) to morphology, attenuation, and size features in CECT was found more accurate than response assessment by RECIST in renal cell carcinoma [9]. CT texture features characterizing the spatial variations of tissue density were shown to be prognostic factors in non-small cell lung cancer (NSCLC) [10] and esophageal cancer [11].

## PET Features

In recent years, FDG-PET imaging, which measures functional (metabolic activity) changes, has shown advantages over anatomic imaging as a response evaluation tool in many malignancies [3,12–16]. For example, in NSCLC [12,17] and esophageal cancer [14,18–22], FDG PET imaging has shown superior results in predicting survival and pathologic response to chemoradiotherapy (CRT) compared with conventional CT imaging. Both the European Organization for Research and Treatment of Cancer (EORTC) [23] and the PET Response Criteria in Solid Tumors (PERCIST) [3] developed guidelines for the methodology of evaluating tumor response with serial FDG-PET, with the goal of achieving standardization in clinical trials. Despite these encouraging results, the reported accuracy for predicting response to CRT using PET/CT is often not high enough (<90%) for clinicians to make critical treatment decisions confidently. For example, a pooled sensitivity of 67% (range: 33% – 100%) and specificity of 68% (range: 30% – 100%) were reported in 20 studies for esophageal cancer [21]. Furthermore, none of these studies have demonstrated both high sensitivity and high specificity.

Almost all of the published (to our knowledge) FDG-PET studies quantify therapeutic response in tumors with *SUVmax* - the maximum standard uptake value (SUV) of FDG within a tumor [24–26]. In these studies, changes in *SUVmax*, or sometimes *SUVmax* before (pre-) CRT or after (post-) CRT, are correlated to post-CRT pathologic response, or survival, or both. *SUVmax* is a single point estimate which ignores changes in the distribution of FDG uptake within a tumor and in the extent of metabolic abnormality. However, it is known that most solid tumors consist of various malignant and non-malignant components so that they show significant heterogeneity in both the degree and distribution of FDG uptake. Heterogeneity in FDG uptake is associated with important biological and physiologic parameters [27–31], and has been shown to be prognostic in many cancers [27,28,30–34]. Another limitation of *SUVmax* is that it exhibits dependence on image noise and image resolution [3,35–37]. Recent studies suggest that new PET/CT features considering spatial information, such as tumor volume [38], tumor shape [33,39], total glycolytic volume [3], and texture features [31–33,40,41] are more informative than *SUVmax* and tumor diameters for the prediction of tumor response. Particularly, Tan et al. demonstrated that comprehensive spatial–temporal ^18^F-FDG PET features (intensity, texture, and shape) were more useful predictors of pathologic tumor response to CRT than conventional SUV measures in esophageal cancer [42].

## Future Works

The new PET/CT response features characterize different properties of a tumor suggesting that they contain complementary information. Therefore, it would be advantageous to combine multiple features instead of traditional response criteria that are based on cutoff values of a single measure [3, =21]. Vaidya et al. showed that multivariable logistic regression improved the prediction of local failure for NSCLC by combining complementary PET and CT features [10]. Zhang et al. constructed support vector machine models using spatial–temporal ^18^F-FDG PET features [42]. With cross-validation, the models achieved 100% sensitivity and 90% specificity for the prediction of pathologic tumor response to CRT in patients with esophageal cancer [43].

Though not widely used in clinic, quite a few new PET tracers, including FLT that measures cell proliferation [44,45], FMISO that measures hypoxia [46, 47], and others [48,49] have brought enthusiasm in the evaluation of tumor response for various disease sites. The aforementioned concepts and methods are generally applicable to any tracer and therapy where PET/CT is used for response evaluation.

Challenges for implementing the new methodology with the more comprehensive PET/CT features include delineating the tumor volume in multi-modality (PET/CT) images, identifying a few features that truly capture biological changes correlated with tumor response for a specific disease and therapy, validating the results in large, multicenter patient datasets, vendor implementation and ultimately clinic acceptance.

## References

[1] Eisenhauer EA, Therasse P, Bogaerts J, Schwartz LH, Sargent D (2009). New response evaluation criteria in solid tumours: revised RECIST guideline (version 1.1). Eur J Cancer.

[2] Erasmus JJ, Gladish GW, Broemeling L, Sabloff BS, Truong MT (2003). Interobserver and intraobserver variability in measurement of non-small-cell carcinoma lung lesions: implications for assessment of tumor response. J Clin Oncol.

[3] Wahl RL, Jacene H, Kasamon Y, Lodge MA (2009). From RECIST to PERCIST: Evolving Considerations for PET response criteria in solid tumors. J Nucl Med.

[4] Zhao B, Schwartz LH, Moskowitz CS, Ginsberg MS, Rizvi NA (2006). Lung cancer: computerized quantification of tumor response--initial results. Radiology.

[5] Goldmacher GV, Conklin J (2012). The use of tumour volumetrics to assess response to therapy in anticancer clinical trials. Br J Clin Pharmacol.

[6] Bruix J, Sherman M, Llovet JM, Beaugrand M, Lencioni R (2001). Clinical management of hepatocellular carcinoma. Conclusions of the Barcelona-2000 EASL conference. European Association for the Study of the Liver. J Hepatol.

[7] Choi H, Charnsangavej C, de Castro Faria S, Tamm EP, Benjamin RS (2004). CT evaluation of the response of gastrointestinal stromal tumors after imatinib mesylate treatment: a quantitative analysis correlated with FDG PET findings. AJR Am J Roentgenol.

[8] Chun YS, Vauthey JN, Boonsirikamchai P, Maru DM, Kopetz S (2009). Association of computed tomography morphologic criteria with pathologic response and survival in patients treated with bevacizumab for colorectal liver metastases. JAMA.

[9] Smith AD, Shah SN, Rini BI, Lieber ML, Remer EM (2010). Morphology, Attenuation, Size, and Structure (MASS) criteria: assessing response and predicting clinical outcome in metastatic renal cell carcinoma on antiangiogenic targeted therapy. AJR Am J Roentgenol.

[10] Vaidya M, Creach KM, Frye J, Dehdashti F, Bradley JD (2012). Combined PET/CT image characteristics for radiotherapy tumor response in lung cancer. Radiother Oncol.

[11] Yip CSP, Davnall F, Kozarski R, Landau1 D, Mason R (2013). CT Tumoral Heterogeneity as a Prognostic Marker in Primary Esophageal Cancer Following Neoadjuvant Chemotherapy.

[12] Mac Manus MP, Hicks RJ, Matthews JP, McKenzie A, Rischin D (2003). Positron emission tomography is superior to computed tomography scanning for response-assessment after radical radiotherapy or chemoradiotherapy in patients with non-small-cell lung cancer. J Clin Oncol.

[13] Benz MR, Czernin J, Allen-Auerbach MS, Tap WD, Dry SM (2009). FDG-PET/CT imaging predicts histopathologic treatment responses after the initial cycle of neoadjuvant chemotherapy in high-grade soft-tissue sarcomas. Clin Cancer Res.

[14] Krause BJ, Herrmann K, Wieder H, zum Büschenfelde CM (2009). 18F-FDG PET and 18F-FDG PET/CT for assessing response to therapy in esophageal cancer. J Nucl Med.

[15] Heron DE, Andrade RS, Beriwal S, Smith RP (2008). PET-CT in radiation oncology: the impact on diagnosis, treatment planning, and assessment of treatment response. Am J Clin Oncol.

[16] Brindle K (2008). New approaches for imaging tumour responses to treatment. Nat Rev Cancer.

[17] Hicks RJ (2009). Role of 18F-FDG PET in assessment of response in non-small cell lung cancer. J Nucl Med.

[18] Westerterp M, van Westreenen HL, Reitsma JB, Hoekstra OS, Stoker J (2005). Esophageal cancer: CT, endoscopic US, and FDG PET for assessment of response to neoadjuvant therapy--systematic review. Radiology.

[19] Swisher SG, Maish M, Erasmus JJ, Correa AM, Ajani JA (2004). Utility of PET, CT, and EUS to identify pathologic responders in esophageal cancer. Ann Thorac Surg.

[20] Levine EA, Farmer MR, Clark P, Mishra F, Ho C (2006). Predictive value of 18-fluoro-deoxy-glucose-positron emission tomography (18F-FDG-PET) in the identification of responders to chemoradiation therapy for the treatment of locally advanced esophageal cancer. Ann Surg.

[21] Kwee RM (2010). Prediction of tumor response to neoadjuvant therapy in patients with esophageal cancer with use of 18F FDG PET: a systematic review. Radiology.

[22] Monjazeb AM, Riedlinger G, Aklilu M, Geisinger KR, Mishra G (2010). Outcomes of patients with esophageal cancer staged with [^18^F] fluorodeoxyglucose positron emission tomography (FDG-PET): can postchemoradiotherapy FDG-PET predict the utility of resection?. J Clin Oncol.

[23] Young H, Baum R, Cremerius U, Herholz K, Hoekstra O (1999). Measurement of clinical and subclinical tumour response using [18F]-fluorodeoxyglucose and positron emission tomography: review and 1999 EORTC recommendations. European Organization for Research and Treatment of Cancer (EORTC) PET Study Group. Eur J Cancer.

[24] Kubota K (2001). From tumor biology to clinical Pet: a review of positron emission tomography (PET) in oncology. Ann Nucl Med.

[25] Cannon BA (2010). Improving Quantitative Treatment Response Monitoring With Deformable Image Registration. UT GSBS Dissertations and Theses.

[26] Larson SM, Erdi Y, Akhurst T, Mazumdar M, Macapinlac HA (1999). Tumor Treatment Response Based on Visual and Quantitative Changes in Global Tumor Glycolysis Using PET-FDG Imaging. The Visual Response Score and the Change in Total Lesion Glycolysis. Clin Positron Imaging.

[27] Aerts HJ, van Baardwijk AA, Petit SF, Offermann C, Loon Jv (2009). Identification of residual metabolic-active areas within individual NSCLC tumours using a pre-radiotherapy (18)Fluorodeoxyglucose-PET-CT scan. Radiother Oncol.

[28] Belhassen S, Zaidi H (2010). A novel fuzzy C-means algorithm for unsupervised heterogeneous tumor quantification in PET. Med Phys.

[29] Zhao S, Kuge Y, Mochizuki T, Takahashi T, Nakada K (2005). Biologic correlates of intratumoral heterogeneity in 18F-FDG distribution with regional expression of glucose transporters and hexokinase-II in experimental tumor. J Nucl Med.

[30] Zhou SM, Wong TZ, Marks LB (2004). Using FDG-PET activity as a surrogate for tumor cell density and its effect on equivalent uniform dose calculation. Med Phys.

[31] Tixier F, Le Rest CC, Hatt M, Albarghach N, Pradier O (2011). Intratumor heterogeneity characterized by textural features on baseline 18F-FDG PET images predicts response to concomitant radiochemotherapy in esophageal cancer. J Nucl Med.

[32] Eary JF, O’Sullivan F, O’Sullivan J, Conrad EU (2008). Spatial heterogeneity in sarcoma 18F-FDG uptake as a predictor of patient outcome. J Nucl Med.

[33] El Naqa I, Grigsby P, Apte A, Kidd E, Donnelly E (2009). Exploring feature-based approaches in PET images for predicting cancer treatment outcomes. Pattern Recognit.

[34] Marusyk A, Polyak K (2010). Tumor heterogeneity: causes and consequences. Biochim Biophys Acta.

[35] Hatt M, Cheze-Le Rest C, Aboagye EO, Kenny LM, Rosso L (2010). Reproducibility of 18F-FDG and 3′-deoxy-3′-18F-fluorothymidine PET tumor volume measurements. J Nucl Med.

[36] Moeller BJ, Rana V, Cannon BA, Williams MD, Sturgis EM (2009). Prospective risk-adjusted [18F]Fluorodeoxyglucose positron emission tomography and computed tomography assessment of radiation response in head and neck cancer. J Clin Oncol.

[37] Boellaard R, Krak NC, Hoekstra OS, Lammertsma AA (2004). Effects of noise, image resolution, and ROI definition on the accuracy of standard uptake values: a simulation study. J Nucl Med.

[38] Prasad SR, Jhaveri KS, Saini S, Hahn PF, Halpern EF (2002). CT tumor measurement for therapeutic response assessment: comparison of unidimensional, bidimensional, and volumetric techniques initial observations. Radiology.

[39] O’Sullivan F, Roy S, O’Sullivan J, Vernon C, Eary J (2005). Incorporation of tumor shape into an assessment of spatial heterogeneity for human sarcomas imaged with FDG-PET. Biostatistics.

[40] Yu H, Caldwell C, Mah K, Mozeg D (2009). Coregistered FDG PET/CT-based textural characterization of head and neck cancer for radiation treatment planning. IEEE Trans Med Imaging.

[41] Yu H, Caldwell C, Mah K, Poon I, Balogh J (2009). Automated radiation targeting in head-and-neck cancer using region-based texture analysis of PET and CT images. Int J Radiat Oncol Biol Phys.

[42] Tan S, Kligerman S, Chen W, Lu M, Kim G (2013). Spatial-temporal [^18^F] FDG-PET features for predicting pathologic response of esophageal cancer to neoadjuvant chemoradiation therapy. Int J Radiat Oncol Biol Phys.

[43] Zhang H, Tan S, Chen W, Kligerman S, Kim G (2012). WE-C-BRA-01: Best in Physics (Joint Imaging-Therapy) - Modeling Pathologic Response of Locally Advanced Esophageal Cancer to Chemoradiotherapy Using Spatial-Temporal FDG-PET Features. Clinical Parameters and Demographics: AAPM.

[44] Herrmann K, Ott K, Buck AK, Lordick F, Wilhelm D (2007). Imaging gastric cancer with PET and the radiotracers 18F-FLT and 18F-FDG: a comparative analysis. J Nucl Med.

[45] Yue J, Chen L, Cabrera AR, Sun X, Zhao S (2010). Measuring tumor cell proliferation with 18F-FLT PET during radiotherapy of esophageal squamous cell carcinoma: a pilot clinical study. J Nucl Med.

[46] Chang JH, Wada M, Anderson NJ, Lim Joon D, Lee ST (2013). Hypoxia-targeted radiotherapy dose painting for head and neck cancer using (18) F-FMISO PET: A biological modeling study. Acta Oncol.

[47] Hicks RJ, Rischin D, Fisher R, Binns D, Scott AM (2005). Utility of FMISO PET in advanced head and neck cancer treated with chemoradiation incorporating a hypoxia-targeting chemotherapy agent. Eur J Nucl Med Mol Imaging.

[48] Lehtiö K, Eskola O, Viljanen T, Oikonen V, Grönroos T (2004). Imaging perfusion and hypoxia with PET to predict radiotherapy response in head-and-neck cancer. Int J Radiat Oncol Biol Phys.

[49] Wieder H, Ott K, Zimmermann F, Nekarda H, Stollfuss J (2002). PET imaging with [11C]methyl-L-methionine for therapy monitoring in patients with rectal cancer. Eur J Nucl Med Mol Imaging.

